# Establishing intersectoral ‘Schools Teams’ to mitigate SARS-CoV-2 school transmission, 2020/2021: Peter Naughton

**DOI:** 10.1093/eurpub/ckac129.283

**Published:** 2022-10-25

**Authors:** C Kelly, P Naughton, E Kennedy, M Ward

**Affiliations:** 1 Health Protection Surveillance Centre, Dublin, Ireland; 2 Department of Public Health HSE East, CHO Area 6, Dublin, Ireland

## Abstract

The SARS-CoV-2 pandemic disrupted the lives of up to 100,000 school-going children in Ireland. Consequently, intersectoral ‘Schools Teams’ were established for the 2020/2021 school year to reduce SARS-CoV-2 transmission in school settings. This novel public health intervention provides learning to inform future cross-sectoral collaborative work in Public Health in responding to infectious disease threats. For the 2020/2021 school year in Ireland, intersectoral Schools Teams were formed within each of eight regional Departments of Public Health to manage mitigation of SARS-CoV-2 transmission in school settings. These teams comprised of staff from Departments of Public Health and redeployed staff from the Department of Education. A nationally agreed schools process was followed by Schools Teams to manage SARS-CoV-2 cases and outbreaks in schools. Relevant cases were referred to the regional Schools Team for a public health risk assessment (PHRA). Close contacts were determined using appropriate definitions of close contact within a school setting through the PHRA. This model with centralised procedures and linked health/education teams was novel and adaptable to additional settings. Results from the East region of Ireland showed testing of close contacts of COVID-19 was conducted in 71.8% (676/942) of schools, with 43881 tests completed. Most Schools Team members reported efficient communication within the team (88.7%), a positive team culture (96.3%) and feeling comfortable in their roles following training (82.7%). The majority of members felt the team was able to effectively support schools to reduce COVID-19 transmission (92.5%). Lessons learnt include the synergistic working of educational and health professionals towards a common goal, maximising the skills of all, ensuring a better outcome for school children. Involving educational teams in active contact tracing of COVID-19 cases in schools maximised engagement of the educational sector in the COVID-19 response.

**Key messages:**

• Establishing intersectoral ‘Schools Teams’ pooled skills, resources and expertise, enabling development of synergistic solutions to a complex problem.

• This exemplifies a large national cross-sectoral collaborative working process involving education and public health sectors, providing a model for future responses to infectious disease threats.

